# Fast detection, a precise and sensitive diagnostic agent for breast cancer

**DOI:** 10.1186/s13046-022-02393-3

**Published:** 2022-06-13

**Authors:** Qiong Wu, Chanling Yuan, Ningzhi Liu, Jing Shu, Jiacheng Wang, Jiayi Qian, Liang Zeng, Hao Zhang, Xicheng Wang, Wenjie Mei

**Affiliations:** 1 School of Pharmacy, Guangdong Pharmaceutical University, Guangzhou, 510006 China; 2 The First Affiliation Hospital of Guangdong Pharmaceutical University, Guangzhou, 510062 China; 3 Guangdong Province Engineering Technology Centre for Molecular Probe and Bio-Medical Imaging, Guangdong Pharmaceutical University, Guangzhou, 510006 China; 4 Department of Pathology, Guangzhou Women and Children’s Medical Center, Guangzhou Medical University, Guangzhou, 510623 China

**Keywords:** AS1411, NCL, Breast cancer, Tumor-targeting imaging

## Abstract

**Background:**

Breast cancer targeting diagnostic agent with effective imaging ability is important in guiding plan formulation, prediction, and curative effect evaluation of tumors in clinic. A tumor-targeting nanoprobe based on the functional and programmable Liquid–Liquid phase separation of AS1411 promoted by Ru(II) complex RuPEP may develop into a potential phosphorescence probe to detect breast cancer cells, where AS1411 act as a tumor-targeting guidance moiety to distinguish tumor cells from normal cells and RuPEP act as a light-emitting element to highlight breast cancer cells.

**Methods:**

Here we designed and constructed a nanoprobe AS1411@RuPEP, and the physicochemical and biochemical properties were characterized by TEM, AFM and EDS. The breast cancer targeting diagnostic capacity was evaluated by normal/tumor cell co-culture assay, tumor cells targeting tracking in xenograft model and cancerous area selectively distinguishing in human patient tissue.

**Results:**

Further studies indicated that the nanoprobe exhibits excellent tumor-targeting imaging ability in vitro and in vivo by effectively recognize the over-expressed nucleolin (NCL) on the breast cancer cells membrane. Intriguingly, we discovered that the selectively enrichment of nanoprobe particles in tumor cells is related to ATP-dependent NCL transport processes that rely on the AS1411 component of nanoprobe to recognize NCL. Furthermore, preferential accumulation of nanoprobe is clearly differentiating the human breast cancer tissue surrounding non-cancerous tissue in histological analysis.

**Conclusion:**

This study produce a potent nanoprobe can be used as a convenient tool to highlight and distinguish tumor cells in vivo, and indicate the tumorous grading and staging in human breast cancer patient pathological section, which provides an effective way for breast cancer diagnostic imaging by targeting recognize NCL.

**Supplementary Information:**

The online version contains supplementary material available at 10.1186/s13046-022-02393-3.

## Background

In current precision medicine and individual medical era, molecular imaging has long been investigated for their potential utility in early accurate diagnosis and staging of tumors, and guiding plan formulation, as well as prediction and evaluation of curative effect [1]. Ascribe to its highly specify to small molecules, aptamers, which are synthetic, short and single-stranded oligonucleotides derived from SELEX methods, have attracted more and more attentions for their potential utility in constructing nanoprobe [2–4]. In general, aptamers can be linked to nanomaterials to form nanoprobe targeting to tumor cells [5–7]. For example, an anti-MUC1 aptamer have been loaded on the surface of AuNPs through a stable Au–S bond to constitute for tumor-targeting drug delivery system [8]. Moreover, an ATP-binding aptamer can be incorporated into a DNA triangular prism to constitute DNA logic device, and developed potential applications in controllable drug release and disease treatment [9]. It’s known that G-rich aptamers, like AS1411, can form G-quadruplex conformation through Hoogesteen hydrogen bond [10, 11], and AS1411 can targeting interact with nucleolin (NCL), which is over-expressed in various tumors and associated with more aggressive tumors resistance to ionizing radiation, easy recurrence and poor prognosis [12, 13]. It’s also reported that constructed from G4 DNA porphyrin can be facilitated to improve the photosensor and hence killed tumor cells under light irradiation [14]. Nevertheless, the utilization of these in clinic still be limited attributed to their low membrane penetration efficiency, high toxicity and sophisticated skill needed.

Recently, transition metal complexes, especially versatile Ru(II) complexes have attracted more and more attentions as promising probe attributed to their unique electron configurations, long-wavelength and long-life phosphorescence [15–17]. Accumulated evidence show that Ru(II) complexes can be facilitated not only as excellent probe of mitochondria, lysosome and endoplasmic reticulum [18, 19], but also can be used to monitor living cells [20, 21]. Ru(II) complexes have been also reported to act as single and two-photon phosphorescent probes through equipping with near infrared (NIR) and long excitation wavelength groups, small phototoxicity, deep penetration depth, and photobleaching reduction [22, 23]. Moreover, Ru(II) polypyridyl complexes have been investigated as promising deep-tissue photosensor which can pass through the tissues up to 16 mm thickness when activated by red light after synthesis [24], and Ru(II) complexes with alkynes can also be utilized to highlight the nucleus efficiently [25], but be short of tumor selectively imaging ability.

**Scheme 1:**
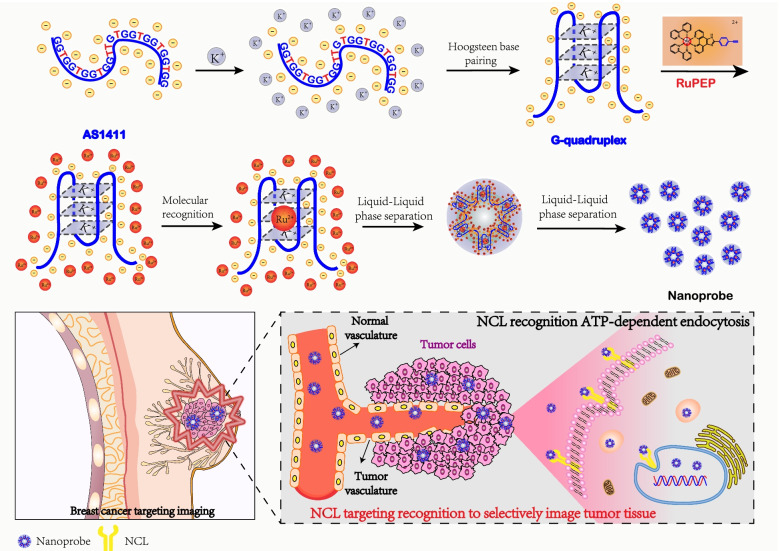
Schematic highlight the constructed nanoprobe of AS1411@RuPEP nanoparticles for precise breast cancer imaging of NCL targeting recognition

With this in mind, a nucleolin-targeting nanoprobe has been constructed by AS1411 interacting with a novel ruthenium(II) complex with alkynles, Λ-[Ru(bpy)_2_(p-EPIP)](ClO_4_)_2_ (RuPEP, bpy = bipyridyl, p-EPIP = 2-(4-ethynyl phenyl)-1H-imidazo [4,5-f] [1, 10] phenanthroline). It’s revealed that RuPEP can interact with the G-quadruplex conformation of AS1411 via groove binding mode, and hence inducing self-assembly of AS1411 to form a nanoprobe through liquid–liquid phase separation (Scheme 1). In such constituted nanoprobe, AS1411 act as tumor-targeting moiety since AS1411 can selectively recognize to NCL on the surface of tumor cells [26, 27], while RuPEP with excellent luminescent property act as phosphorescence probe to highlight tumor cells. After treated with the constructed nanoprobe, breast cancer MDA-MB-231 cells were distinguished by red phosphorescence in the nuclei of tumor cells in the co-culture system with human normal MCF-10A cells. The further studies show that this nanoprobe can image tumor area in nude/Balbc mice bearing MDA-MB-231 tumor cells. Moreover, the nanoprobe has been utilized to successfully distinguish tumor area in breast cancer samples and even to preliminarily assess the tumor grade, which the higher degree of malignancy, the expression of NCL more prosperous. These studies in the underlying mechanism show that the constructed nanoprobe can targeting recognize the nucleolin aggregated on the membrane surface of tumor cells, and can be taken by a process of endocytosis and localize in the nuclei of tumor cells.

## Materials and methods

All reagents and solvents were purchased commercially and were used without purification unless specifically noted. Distilled water was used in all experiments. The novel chiral ruthenium(II) complex RuPEP were prepared in our lab, and the related synthetic route (Figure S1) and characterization data (Figure S2) were listed in Supporting Information. The AS1411 oligonucleotide (sequence 5'-GGTGGTGGTGGTTGTGGTGGTGGTGG-3') was purchased from Sangon Biotech (Shanghai).

### Construction and characterization of nanoprobe AS1411@RuPEP

AS1411 formed a G-quadruplex conformation in Tris–HCl KCl buffer by renaturation at 4 °C for 24 h after 95 °C denaturation for 5 min. Aqueous solutions of AS1411 (50 μM in Tris–HCl KCl buffer) and RuPEP (50 μM in Tris–HCl KCl buffer) were mixed in equal volumes and incubated at 37 °C for three days. The final samples were obtained by dialysis of the mixed solutions for overnight. All biological experiments were performed by using fresh self-assembled nanoprobe after dialysis, vacuum freeze drying and quantified the concentration of Ru atom. To obtain samples for microscopy, aliquots were removed from the mixed solution at a volume of 100 μL and added to a copper mesh TEM grid whereupon the solvent was evaporated for 2 h at room temperature. Images of the sample were then obtained by using transmission electron microscope (TEM) (TECNAI 10, FEI, American) and field emission transmission electron microscope (FETEM) (TECNAI G2 F20, FEI, American). In addition, a portion of sample was extracted at a volume of 10 μL and transferred to a mica plate where solvent was left to volatilize for 2 h. The surface structure was imaged by an AFM (Bruker, Dimension Fast ScanTM, American).

### Cell culture

MDA-MB-231, MCF-7, MCF-10A and GFP-actin labeled MCF-10A cells were cultured in Dulbecco’s modified Eagle medium (DMEM, Hyclone, SH30022.01, American) containing 10% fetal bovine serum (FBS, Siji Green, 3011–8611 China), 10 KU/mL Penicillin–Streptomycin Solution (BioSharp, BL505A, China). GFP-actin labeled MCF-10A cells were obtained through transfered GFP-actin plasmid DNA. The cells were cultured at 37 °C in a humidity incubator supplied with 5% CO_2_. For all experiments, cells were disrupted using 0.25% trypsin (BioSharp, BL527A, China) and were re-plated in fresh medium for subsequent experiments.

### Selectively imaging tumor cells in co-culture assays

We co-cultured MDA-MB-231 and GFP-MCF-10A cells in confocal dishes with microscope slides to investigate whether the nanoprobe could selectively recognize tumor cells and differentiate these from normal human epithelial cells. Normal GFP-MCF-10A epithelial cells were labeled with a green fluorescence marker using transfected GFP-actin plasmid DNA in 3.5 cm dishes plated at a density of 1 × 10^4^ cells/dish. After MCF-10A cells had adhered to the dishes, MDA-MB-231 cells were added to the same dishes at the same density, and all cells were labeled with a blue fluorescence marker by Hoechst 33,258 (BioSharp, BL804A, China). The cells were then incubated with nanoprobe (5 μM) at 37 °C for 6 h [28]. The images from multiple dishes were obtained by the confocal microscopy (Zessis, LSM800, Germany).

### NCL-mediated endocytosis by bio-TEM

To study the cellular uptake process of nanoprobe in live cells, MDA-MB-231 cells were cultured with nanoprobe that had been dispersed in DMEM media (the concentration of nanoprobe was 5 μM) under culture conditions (5% CO_2_, 37 °C) for 24 h. The cells were harvested by cell scraper to ensure that the cells maintained their natural morphology. Subsequently, the collected cells were fixed and placed onto copper grids for bio-TEM observation according to the standard sample preparation procedures for TEM (TECNAI 10, FEI, American).

### Western blotting

MDA-MB-231, MCF-7 and MCF-10A cells (at a density of 5 × 10^4^ cells/mL) were seeded onto cover slips (10-cm diameter) and were allowed to overgrow for 80%, respectively. Five cryopreserved tumor and normal biopsy specimens of invasive ductual carinoma patients were provided by the first affiliated hospital of Guangdong Pharmaceutical University. Total proteins were extracted by incubating cells in RIPA Lysis Buffer (BioSharp, BL504A, China) and protein concentrations were determined by BCA Protein Assay Kit (BioSharp, BL521A, China). SDS-PAGE was done in 10% tricine gels loading equal amount of proteins per lane. After electrophoresis, separated proteins were transferred to nitrocellulose membrane and blocked with 5% non-fat milk in TBST buffer for 1 h. After then, the membranes were incubated with primary antibodies at 1:1000 dilutions in 5% non-fatmilk overnight at 4 °C, and then secondary antibodies conjugated with horseradish peroxidase at 1:10,000 dilution for 1 h at room temperature. The mouse-derived anti-GAPDH antibody (60,004–1-Ig), rabbit-derived anti-NCL antibody (10,556–1-AP), rabbit-derived anti-LBR antibody (12,398–1-AP), rabbit-derived anti-Her2 antibody (18,299–1-AP) were obtained from Proteintech (Wuhan, China). The rabbit-derived anti-Estrogen Receptor alpha (ERα) (ab108398) was obtained from Abcam (Cambridge, UK). IRDye 680 anti-mouse and IRDye 800 anti-rabbit secondary antibodies were obtained from LI-COR (Odessay, American). Protein bands were visualized on Odyssey imaging system (LI-COR Odessay, American).

### Immunofluorescence

MDA-MB-231, MCF-7 and MCF-10A cells (at a density of 5 × 10^4^ cells/mL) were seeded onto cover slips (35-mm diameter) and were allowed to adhere for 12 h. MDA-MB-231, MCF-7 and MCF-10A cells were cultured in the presence of either nanoprobe (0 and 5 μM) at 37 °C for 6 h. Cells were washed once in PBS, fixed, and permeabilized simultaneously using 4% paraformaldehyde with 1% Triton X-100 in PBS. They were then quenched with 0.1 M glycine in PBS, and blocked overnight at 4 °C with 3% (wt/vol) BSA. The fixed and permeabilized cells were stained with primary and secondary antibodies as has been prescribed [27]. Then, cells were stained by DAPI solution (BioSharp, BS097, China) at room temperature for 5 min. Goat Anti-Mouse IgG H&L (Alexa Fluor® 647) (ab150115) and Goat Anti-Rabbit IgG H&L (Alexa Fluor® 488) (ab150077) were obtained from Abcam (Cambridge, UK). Cell morphology was observed using a laser confocal microscope (Zessis, LSM800, Germany).

### Transgenic MMTV-PyMT primacy breast cancer mice

Twenty-four-week old female transgenic MMTV-PyMT primacy breast cancer mice (25–30 g) were purchased from Changzhou Cavens Laboratory Animals Co.; LTD. (Changzhou, China). The mice were maintained in a specific pathogen-free conditions under 12 h lighting/dark cycle and fed with germfree food and water. All in vivo experiments were performed under the guideline approved by the Guangzhou Institute of Biochemistry and Cell Biology, Chinese Academy of Sciences.

### In vivo bioimaging

To assess the tumor-targeting efficacy and image quality of different time regimens of the nanoprobe in vivo, three MMTV-PyMT (B6.FVB-Tg(MMTV-PyVT)634Mul/LellJ) primacy breast cancer mice were intravenously injected with pure saline (100 μL) and used as the control group. Another three mice were intravenously injected with the equivalent nanoprobe dose of 20 μM (100 μL), respectively. All animals were monitored by NIR imaging at 0, 2, 4, 6, 8, 12, 24, 48, 72 and 108 h. Tumor nodules and organs (heart, liver, spleen, lung, kidney and brain) were removed at 24, 72 and 108 h, respectively, and ex vivo NIR imaging was performed.

### In vitro imaging and histological analysis of human breast cancer tissue

The human breast cancer paraffin section (normal specimens and grade-I, grade-II and grade-III specimens of invasive ductal carcinoma patients, each group of ten cases) were provided by the first affiliated hospital of Guangdong Pharmaceutical University and Guangzhou Women and Children's Medical Center. All experiments were conducted under guidelines approved by the ethics committee of the first affiliated hospital of Guangdong Pharmaceutical University. The frozen sections (OCT-embedded) of the fresh human invasive ductal carcinoma tissues were dried by cool wind and washed once by PBS. Then, the frozen sections were permeabilized by 0.5% Triton X-100 PBS solution and blocked 1 h at room temperature with 5% (wt/vol) BSA. The blocked and permeabilized tissues were incubated with nanoprobe (5 μM, 20 μL) at 37 °C for 30 min. The sections stained with NCL primary antibody and secondary antibody at 37 °C for 60 min, respectively. However, the paraffin sections were heated at 65 °C for 2 h, dewaxed by xylene concentration gradient (100% xylene twice, 50% xylene once,), dehydrated by ethanol concentration gradient (100% ethanol twice, 95% ethanol once, 90% ethanol once, 80% ethanol once, 70% ethanol once). The sections were washed three times with PBS, and microwave antigen retrieval performed by sodium citrate buffer (10 mM Sodium Citrate, 0.05% Tween 20, pH 6.0) for 10 min. They were then washed with 0.1 M glycine in PBS, permeabilized by 0.5% Triton X-100 PBS solution and blocked 1 h at room temperature with 5% (wt/vol) BSA. The blocked and permeabilized tissues were incubated with nanoprobe (5 μM, 20 μL) at 37 °C for 30 min. The sections stained with NCL primary antibody and secondary antibody at 37 °C for 60 min, respectively. Then, The sections examined with a fluorescence microscope (Leica DMI8 Microsystems, Germany) and a laser confocal microscope (Zessis, LSM800, Germany).

### Statistical analysis

Statistical analysis was performed using GraphPad Prism 7.04 software (GraphPad, American). The results are presented as the means ± standard deviations (SDs). The difference between two group was analyzed by a two-tailed t test, while values were compared among multiple groups using one- and two-way ANOVA, respectively. Dunnett's multiple comparisons test was applied to analyze the correlation between control group with sample group. Differences were considered statistically significant when *p* < 0.05 (*), *p* < 0.01(**), *p* < 0.001(***), ns—not significant.

## Results

### Characterization of the constructed DNA nanoprobe

**Fig. 1:**
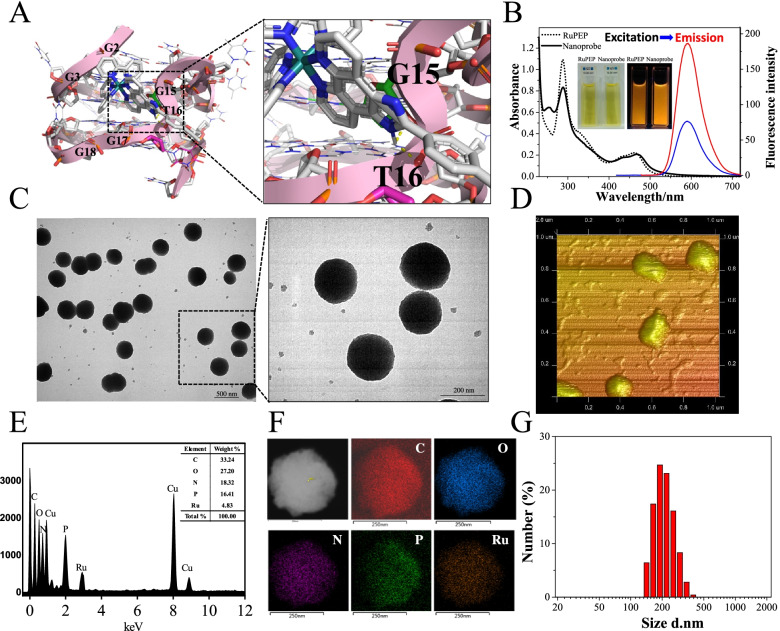
The nano-structure characterization of nanoprobe AS1411@RuPEP. **A** The binding properties of RuPEP interact with AS1411 by molecular docking analysis. **B** The elctronic absorption spectra and fluorescence emission spectra of RuPEP (5 μM) and AS1411@RuPEP (5 μM) in PBS solution. TEM image (**C**) and AFM image (**D**) of AS1411@RuPEP. The EDS analysis (**E**) and the EDS mapping (**F**) of elemental spectrum of AS1411@RuPEP. **G** Number-average diameters and particle size of AS1411@RuPEP in PBS, measured by DLS

In the macroscopic world, nanoprobes are assemblies of components designed to achieve a specific function. Each component of the assembly performs a simple action, while the entire assembly performs a more complex, useful function that is characteristic of that particular device or machine. AS1411 could form stable G-quadruplex structures in the presence of K^+^ and specifically bind to nucleolin on the tumor cells membrane [10, 11]. In the previous study, we screened a chiral Ru (II) complex (laevoisomer) with alkyne, which exhibited strong affinity to AS1411 G-quadruplex DNA in groove binding mode through π-π stacking, and it is observed that two intramolecular hydrogen bond formation between N atom in midazole ring of RuPEP with two H atoms of G15 and T16 residues by molecular docking (Fig. 1A). Here, the construction of this nucleolin-targeting nanoprobe is achieved through liquid–liquid phase separation of aptamer AS1411 and anchoring a phosphorous chiral ruthenium(II) complex RuPEP [29]. Moreover, it is observed that the nanoprobe displayed stronger fluorescence than equimolar RuPEP, which may be attributed to switch-on assay of RuPEP interacting with AS1411 to enhance the fluorescence emission of nanoprobe (Fig. 1B) [30]. After initial assembly of nanoprobe, a high-order structural re-arrangement was observed by TEM (Fig. 1C). Observably, the monodispersed nanoparticles with average diameter of 200 nm were seen for composites extracted from solution, the proposed nanoparticle structures that spontaneously assembled were also supported by AFM observations that showed well-dispersed, uniform size distribution with a mean diameter of 200 nm (Fig. 1D).

We couple elemental mapping using energy-dispersive X-ray spectroscopy in a transmission electron microscope (TEM-EDS) with colocation analysis to study the elemental distribution and the degree of homogeneity in the nanoprobe. The elemental composition analysis employing EDS showed the presence of a strong signal from the P atoms (16.41%) contributed to AS1411 molecules, and a obvious signal from the Ru atoms (4.83%) affiliated to RuPEP (Fig. 1E). Moreover, other obvious peaks for other elements C (33.24%), N (18.32%), and O (27.20%) that from AS1411 and RuPEP observed. Above EDX analysis suggesting the assembly of AS1411 and RuPEP constructed nanoprobe successfully. Furthermore, the elemental maps clearly demonstrate that the C, P and Ru elements are not distributed homogeneously. P and Ru are more majorly centralized at the particle core while C are preferentially found closer to the particle surface. We observe similar properties throughout the sample, with strong spatial correlation between P and Ru, and the enrichment of two atoms in the particle centre (Fig. 1F). In addition, the nanoprobe exhibited mean lengths ranging from 200–500 nm that was confirmed by data from a Malvern laser particle analyzer, showing multiple nanoprobe bound structures (≈ 200 nm, Fig. 1G) [31]. After binding to RuPEP, the zeta potential of AS1411 increased negatively by almost double the initial value, indicating that the NPs became more colloidally stable than free AS1411 particles with amorphous structure (Figure S3A) [32]. To further confirm the stability of the nanostructures in solution, the changes of nanometer size was monitored by a Malvern laser particle analyzer over time. We found that the size of nanoprobe increased slightly with the increasing time from 2 to 72 h, and stabilized to about 200–500 nm after 72 h (Figure S3B). We regarded this as evidence that the nanoprobe exhibits some degree of stability over time in aqueous solutions.

In addition to observe the aggregation of AS1411 in the presence of RuPEP in the aqueous solution by laser diffraction we used analytical ultracentrifugation (AUC) to evaluate molecular weight accretion as a function of time. The results are shown in Figure S4(in supporting information). The nanoprobe accretion complex displayed three possible states having the molecular weights of 47.3, 75.8, and 131 kDa, in which the corresponding sedimentation coefficients (SC) were about 3.5 S (polymer), 4.5 S (polymer), and 9.5 S (polymer), respectively Figure S4(in supporting information). These structures are larger than those of free AS1411, which exhibited two possible states with molecular weights of 27.9 kDa (SC is about 3.2 S) and 43.0 kDa (SC is about 4.2 S) [33]. After prepared nanoprobe for 72 h, increasing sedimentation coefficients and molecular weights were observed for AS1411 after the addition of RuPEP. These data indicate that in the presence of equimolar concentrations of RuPEP, more rigid, high-order nanoparticles structures self-assemble from random coil AS1411 [34].

### Cellular uptake and elevated localization of nanoprobe in nuclei of tumor cells

**Fig. 2:**
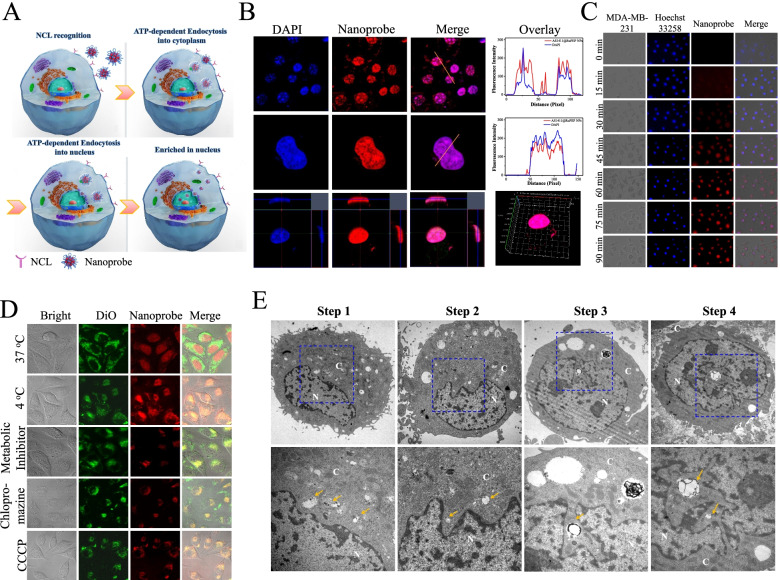
Nanoprobe highlight tumor cells nucleus through ATP-dependent endocytosis. **A** Schematic illustration for the process of nanoprobe entered into nucleus by endocytosis. **B** The cellular localization of AS1411@RuPEP (5 µM) in MDA-MB-231 cells. The enlarge and 3D tomoscan image of the MDA-MB-231 nucleus in the presence of AS1411@RuPEP. The transport pathway of cellular uptake for complex AS1411@RuPEP in MDA-MB-231 cells. **C** Real-time imaging of the MDA-MB-231 cells treated with complex AS1411@RuPEP (5 μM) for 2 h. The cell morphology was captured using phosphorescence microscopy every 15 min. **D** Celluar uptake of AS1411@RuPEP (5 μM) was incubated in MDA-MB-231 cells labeled cell membrane in green fluorescence by DiO for 6 h at 37 °C and 4 °C; MDA-MB-231 cells cells were pre-treatment with Chlorpromazine (6 nM); Dexy (10 nM) + Olig (5 nM), CCCP (10 nM), respectively, then replacement of the inhibitors with AS1411@RuPEP (5 μM) was incubated in MDA-MB-231 cells for 6 h at 37 °C. **E** Bio-TEM imaging of MDA-MB-231 cells for cellular uptake of AS1411@RuPEP. Cells are treated with AS1411@RuPEP for 6 h at 37 °C

Then, we used NCL high-expression breast cancer MDA-MB-231 cells to study the tumor-targeting recognition ability of the nanoprobe. After incubation in MDA-MB-231 breast cancer cells, nanoprobe is completely absorbed by the cells and emits strong red phosphorescence from the cell nuclei (Fig. 2B). We observed that the bright red phosphorescence (nanoprobe) co-localized at the same site and completely overlaid the blue fluorescence band. In the magnified images, two-color fluorescence bands were confined to the cell nuclei. The overlap ratio of the three color bands originating from nanoprobe and DAPI was very close to 100%. In addition, the red fluorescence in 3D tomoscan imaging from depth sectioned images filled the entire nucleus and matched the staining pattern observed for nanoprobe and DAPI. These results indicated that nanoprobe were efficiently absorbed and retained by tumor cells and localized in the nuclei of these cells.

To ascertain the cellular uptake mechanism of nanoprobe for nuclear translocation from extracellular environment to nucleus (Fig. 2C and D) [35], we cultured MDA-MB-231 cells with cell membrane probe DiO for 30 min, when cell membrane were labelled green fluorescence, the cells were treated with nanoprobe (5 μM) at either 37 °C or 4 °C for 6 h. The majority of nanoprobe localized in the nucleus and displayed a little overlay with green DiO when incubated at 37 °C, whereas the nanoprobe remained in the cell cytoplasm or membrane displayed perfect overlay with green DiO when incubated at 4 °C. Based on above results, we hypothesize that nanoprobe enters the cell nucleus through an energy-dependent pathway deriving from an active transport mechanism that drives NCL to the nucleus by intra-cytoplasmic trans-localization. These processes are slowed at 4 °C. Usually, endocytosis describes an energy-dependent process for a general entry mechanism for various extracellular materials. In this process clathrin-coated pits are the primary plasma membrane specialization vehicle involved in the uptake of a wide variety of molecules [36, 37]. To clearly confirm the specific endocytotic pathway involved in cellular internalization of nanoprobe, we pretreated MDA-MB-231 cells with chlorpromazine (clathrin-dependent inhibitor) for 1 h before incubation with nanoprobe. We then observed that the fluorescence signals from nanoprobe mainly localized at the cell surface membrane with great merge version with green DiO (Fig. 2D). These data suggested that nanoprobe are processed by living cancer cells through an endocytotic pathway [38]. Initially, 2-deoxy-D-glucose and oligomycin, which is a common inhibitor combination acting as an ionophore that reduces the ability of ATP synthesis to function optimally, were employed to determine the mechanism underlying essential nuclear accumulation [39]. Interestingly, cells treated with 2-deoxy-D-glucose and oligomycin exhibited significant inhibition of staining by nanoprobe in the nucleus (Fig. 2D). Again, this data supports the view that the uptake of nanoprobe into the nucleus is mainly caused by an energy-dependent active transport pathway.

Bio-TEM was performed to shed more light on the cellular uptake of nanoprobe in breast cancer cells. After incubation with nanoprobe, MDA-MB-231 cells were harvested and sectioned for bio-TEM analysis [40]. As shown in Fig. 2E, nanoprobe were trapped inside vesicles that were observed in the cytoplasm and nucleus. It is observed that nanoprobe may induce the MDA-MB-231 cells to produce several vesicles to carry them entered the cytoplasm and moved near nuclear envelope (yellow arrow in Fig. 2E, step 1 and step 2). Numerous nanoprobe complexes with different sizes and shapes were found in these vesicles, which are illustrated (yellow arrows). The vesicles containing nanoprobe particles gradually approached the nucleus and their contact with the nuclear membrane appears to have triggered its disruption (step 2), after which the particles enter the nucleus through ATP-dependent endocytosis (step 3). Images of the nanoprobe complex escaping from vesicles are shown by pointing red arrows in cell the nucleus (step 4). The escape from vesicles is an important function for completion of their multi-faceted operation. We assume that the nuclear distribution of nanoprobe particles is related to ATP-dependent NCL transport processes that rely on the AS1411 component of nanoprobe to recognize and bind to NCL.

### Imaging tumor cells by the constructed nanoprobes through targeting recognize nucleolin

**Fig. 3:**
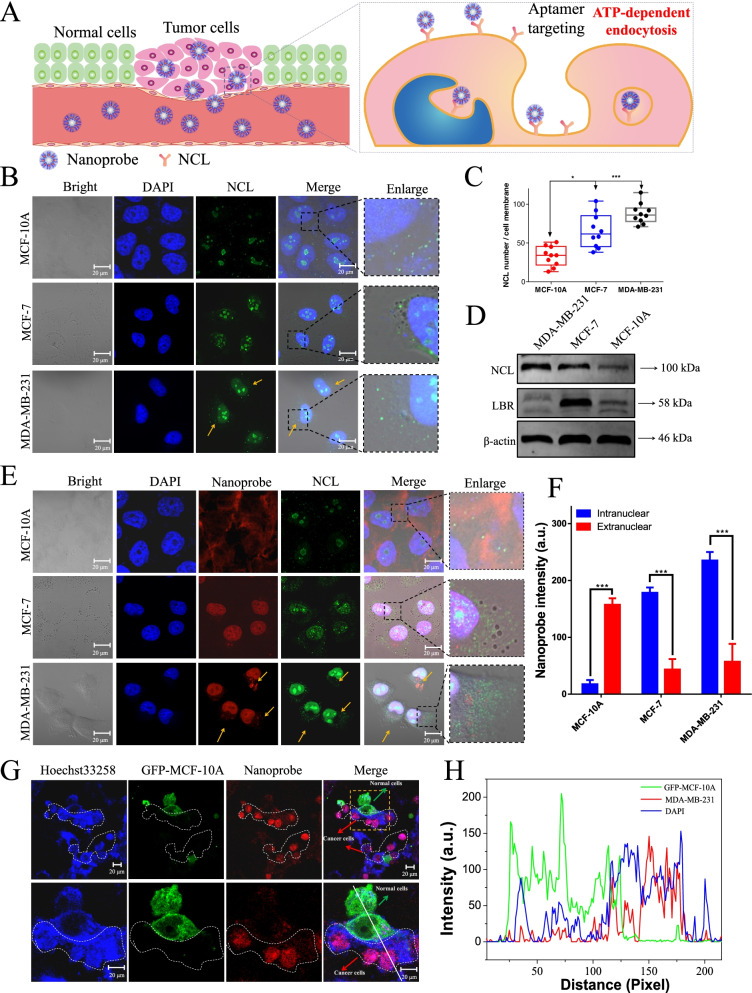
Tumor-selective imaging of NCL targeting recognition by AS1411@RuPEP nanoprobe. **A** The possible process of AS1411@RuPEP nanoprobe targeting recognize NCL on the cells membrane surface to selective image tumor cells. The distribution (**B**) and expression (**C** and **D**) of NCL in breast cancer MDA-MB-231, MCF-7 cells and human normal MCF-10A cells. The localization (**E** and **F**) of AS411@RuPEP nanoprobe in breast cancer MDA-MB-231, MCF-7 cells and human normal MCF-10A cells. **G** LSCM images on co-cultivation of MDA-MB-231 and MCF-10A cells in one confocal dish after incubation with 0.2 mL AS1411@RuPEP (5 μM) for 6 h. Scale bar = 20 μm. **G** The overlay data were analyzed using Image Pro Plus

NCL is a major nucleolar protein that is able to shuttle between the cell surface, the cytoplasm, and the nucleus- a property that makes NCL an attractive target for the selective delivery of anti-tumor drugs without affecting normal cells [41]. A number of studies indicated that NCL is over-expressed in human breast cancer cells and largely distributed on the surface of the cell membrane [10, 11]. However, in normal epithelial cells, NCL is mainly confined within the cell nucleus and deficient in cell membrane [10, 11]. As shown in Fig. 3B, in three cases, it is clearly located in the nucleoli that perfectly matched DAPI staining cell nucleus and non-staining nucleoli. It is confirmed that NCL is mainly distributed in cell nucleolus and only a little in cell membrane, as well as is abundant in tumor cells. Literature reported NCL is over-expressed (from three to six fold increase) in human breast cancer cell lines compared with normal cells [42]. And then, further study shown that the expression of NCL in MDA-MB-231 is obviously higher than in MCF-7 cells, and in MCF-10A cells is prominent deficient (Fig. 3C and D). Moreover, according to the analysis of NCL expression on transcription level with patients’ survival in breast cancer using GEPIA2 and Kaplan–Meier plotter based ATGC database [18]. The data also shown that NCL is higher expression in BRCA (breast cancer) patients tissue than normal tissue (Figure S6), the patients with high expression of NCL also exhibited poorer survival outcomes.

We were curious to examine the distribution of nanoprobe in the cytoplasm of cancer cells to see if they should distribute intracellularly in the absence of metabolic transport. For MCF-10A cells, nanoprobe fails to enter the cells (Fig. 3E), NCL targets in normal epithelial cells exhibit weak and diffuse uptake. But for MDA-MB-231 and MCF-7 cells in the presence of nanoprobe (red phosphorescence, Fig. 3E) was overlaid by DAPI stain in the cell nucleus, and the number of NCL loci shows a significant increase than MCF-10A cells (Fig. 3F). These results suggested that nanoprobe may selectively recognize and activate transport of cell surface NCL receptors to cell nuclei and thereby facilitate imaging of breast cancer cells.

To further evaluate the selectivity of the nanoprobe for breast cancer cells, we developed a cell culture model in which MDA-MB-231 and MCF-10A cells were co-cultured on microscope slides. Considering that NCL is over-expressed in MDA-MB-231 human breast cancer cells and is deficient in MCF-10A normal immortalized human epidermal cells, it should be expected that uptake of the nanoprobe should preferentially localize in the breast cancer cell line. To differentiate between the two cell lines in co-culture, we used MCF-10A cells with green fluorescence through GFP-labeled actin. All cells in co-culture system were labeled with blue fluorescence by Hoechst 33,258. We incubated nanoprobe with the co-cultures at 5 μM for 6 h. Fig. 3G shows strong red phosphorescence in the nucleus of MDA-MB-231 cells, while only a feeble red phosphorescence was observed in MCF-10A cells. These results clearly demonstrate that nanoprobe specifically target and identify tumor cells in mixed cultures.

### In vivo imaging tumor cells

After confirming that the probe selectively binds and translocates as expected to breast cancer cells in culture, we investigated its performance in vivo intransgenic MMTV-PyMT primacy breast cancer mice. The MMTV-PyMT transgenic murine model of breast cancer is a well characterized model which recapitulates human disease, with progression from hyperplasia to invasive carcinoma and metastatic disease at ~ 115 days of life [43]. Then, the transgenic strain MMTV-PyMT mice is a great in vivo model for the study of mammary carcinoma formation and progression with important clinical utility. Here, 24 weeks old MMTV mice (*n* = 3) were used to assess the specific tumor-targeting imaging capability of nanoprobe at different tail intravenous injection time points. According to Figure S10, it is observed that there are multiple tumors under neck, armpit and groin area, some tumor size even over 1 cm.

**Fig. 4:**
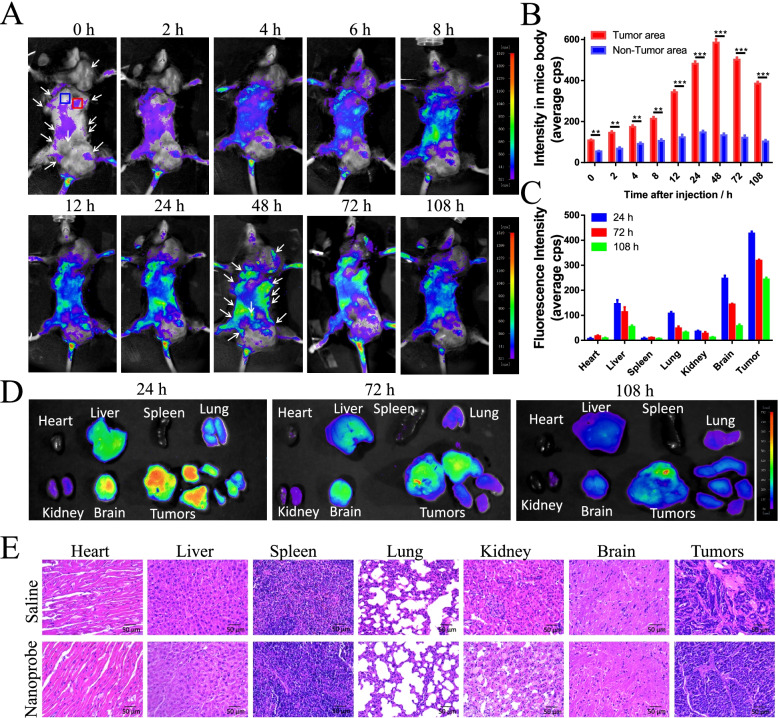
Specific-targeted NIR fluorescence tumor imaging intransgenic MMTV-PyMT primacy breast cancer mice. **A** The arrows show the tumor sites. Time-dependent in vivo NIR fluorescence images of transgenic MMTV-PyMT primacy breast cancer mice after tail intravenous injection of 20 μM, 100 μL, AS1411@RuPEP. (Excited by 475 nm, emission at 680 nm). **B** Fluorescence intensity of AS1411@RuPEP in mice tumor area and non-tumor area is quantitatively determined. **C** Fluorescence intensity of AS1411@RuPEP (average cps) in dissected organs or tissues is quantitatively determined. Data are presented as the mean ± SD (*n* = 3). *p < 0.05, **p < 0.01, ***p < 0.001. **D** Tissue distribution and drug metabolism of AS1411@RuPEP at 24, 72 and 108 h. **E** Histochemistry analysis of heart, liver, spleen, lung, kidney and brain section stained with hematoxylin eosin of transgenic MMTV-PyMT primacy breast cancer mice at 108 h after i.v. administration of saline and 20 μM. Bar: 50 μm

The MMTV-PyMT mice were unhaired by using depilatory paste. When AS1411@RuPEP are intravenously injected into MMTV-PyMT mice, the fluorescence intensity in tumor significantly increases with prolonged circulation time, indicating that AS1411@RuPEP can serve as a promising tumor targeting imaging agent with high contrast for tumor fluorescence imaging. Tumor areas were well defined in the mice within the first 6 h as the nanoprobe rapidly recognizes and binds its NCL targets in tumor tissues, probably because AS1411@RuPEP with superior long-term dispersion stability was easy to pass through the blood vessel and enriched at the tumor site (Fig. 4A). But the two biggest tumors are weakly imaged, it is found that these tumors are necrosis and gangrenosis after dissection. Then, the nanoprobe could not highlight necrotic tumor tissue resulted from lacking a regular metabolism [44]. And the peak fluorescence signal of AS1411@RuPEP in tumor occurs at 48 h post injection, but the fluorescence intensity decreased gradually with the increasing time (72 and 108 h) (Fig. 4B). The result indicates that the NCL-targeted AS1411@RuPEP can be efficiently accumulated and detained in tumor tissues, which could be attributed to the interaction of AS1411 with NCL over-expressed by tumor cells. By observing the fluorescent pictures of the mice’s internal organs and tumors, it was found that AS1411@RuPEP mainly accumulated in brain and tumor, a small concentration of AS1411@RuPEP in liver and lung at 24 h post injection. (Fig. 4C and D). Moreover, the fluorescence intensity of nanoprobe in liver, brain and tumors decreased obviously by prolonging the time from 24 to 108 h post injection, suggesting that NCL-targeted AS1411@RuPEP can be excreted by the renal pathway.

Owing to the nanoprobe could accumulate in multiple mice organs, considering the possible risk to limit the future clinical application, H&E staining was used to evaluate the damage situation of mice organs after treated with nanoprobe for 108 h. The main organs including the heart, liver, spleen, lung, kidney, brain and tumors were collected for histology analysis. In the H&E assay, the nuclei were stained with hematoxylin as blue and the cytoplasm was stained with eosin as red. Comparing to the control group, there are no noticeable inflammatory lesions or organ damages in the treatment group (Fig. 4E). These results suggested that this nanoprobe exhibited little damage to various organs within a short time.

### In vivo preliminary safety evaluation

The unforeseen side-effect of metal-materials for application in biomedicine is always a major concern. For safety's sake, we evaluated systematic toxicity of nanoprobe in healthy kunming mouse after tail intravenous injection for nanoprobe at a dosage of 50 mg/kg per days for three day. Then, primary tissues (containing heart, liver, spleen, lung, kidney and brain) were histopathologically observed under light microscope by H&E staining (Figure 8E). Compared with the control group, no death and serious body weight loss were found in all test groups during the study period. Major tissues including brain, heart, liver, spleen, lung and kidney have no obvious histopathological abnormalities or lesions in the two groups [45]. These results indicated that multiple dosing of nanoprobe had minimal impact in these tissues, showing that there was no significant side-effect caused by this nanoprobe. But in order to improve the potential application of this nanoprobe in clinic, the long-term toxic effects should be further investigate in the future study.

### Human breast cancer section imaging

**Fig. 5:**
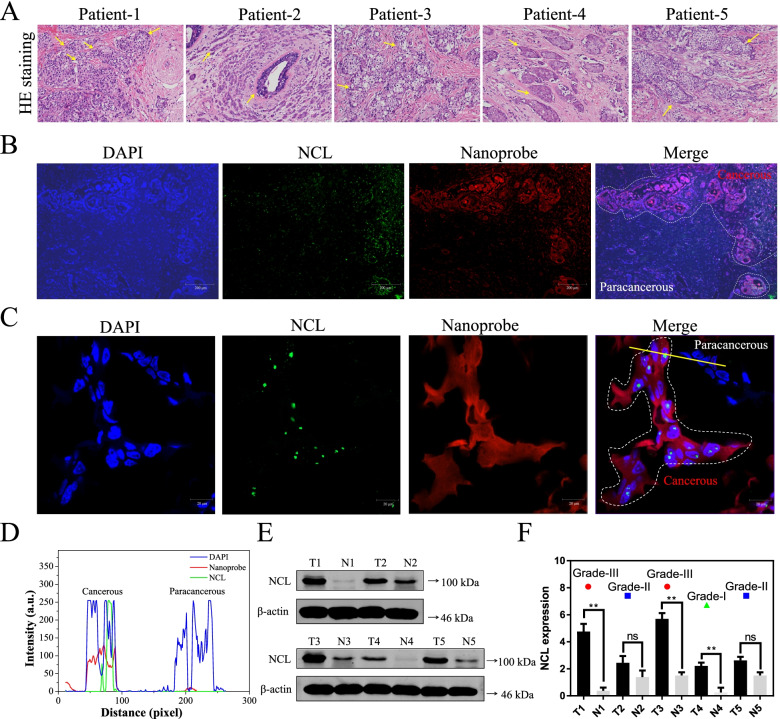
Distinguish and detect tumor area in human breast cancer patient section for nanoprobe. **A** Histochemistry analysis of human breast cancer tissues section stained with hematoxylin eosin of five specimens of invasive ductal carcinoma patients. **B** The targeting imaging of nanoprobe (5 μM) to distinguish cancerous area and paracancerous area in the frozen sections (OCT-embedded) of the fresh human invasive ductal carcinoma tissues observed by fluorescence microscope. **C** CLSM enlarged observation of the distribution of nucleolin and nanoprobe in cancerous area and paracancerous area. The whole tissues were stained by DAPI in blue, NCL stained in Green and Nanoprobe is red. **D** The merge curve of three channels emission intensity for cancerous and paracancerous cells analyzed by using Image-Pro Plus softwire. **E** The NCL protein expression of cancerous and paracancerous tissues in five specimens of invasive ductal carcinoma patients (*n* = 5). **F** Quantitative analysis of NCL expression and tumor grade of five specimens of invasive ductal carcinoma specimens (*n* = 5). Note: The fresh human cancer tissues were The data are presented as the means ± SDs of three independent experiments. *P < 0.05,** *P* < 0.01 and ns—not significant

To further investigate the potential application of nanoprobe as a diagnostic agent for breast cancer in clinical tissue specimens, we used five fresh biopsy specimens of patients with invasive ductal carcinoma of the breast to evaluate the availability of nanoprobe for targeting NCL to image tumor tissue. Histology in the resection specimen revealed that obvious neoplastic lesion was composed of large polygonal cells arranged in infiltrating solid and micropapillary formations, with abundant eosinophilic, vacuolated, and foamy cytoplasm. In situ areas of the lesion contain cells arranged in an alveolar pattern with a hobnail appearance (Fig. 5A) [46]. Also, it is apparent that a segmentation is produced for most of the nuclei in the image, with few contours corresponding to non-epithelial nuclei objects. However, there are clear differences between tumorous and paracancerous tissues. It is observed that the tumor cells are disorganized with incompact structure and deep-dyed bigger nucleolus than normal cells. As mentioned before, the preferential tumor accumulation is considered to be caused by NCL-mediated active transport of nanoprobe. Although the contribution of nanoprobe to tumor accumulation is clear in co-culture system of MDA-MB-231 and MCF-10A cells lines, the potential effectiveness of nanoprobe for targeting tumor imaging is unclear in human breast cancer biopsy specimens.

In histological analysis for the ex vivo tumor samples shown in Fig. 5B, visible blue fluorescence is observed at DAPI mode in the pathological section. Moreover, there is a distinct demarcation between cancerous area with high-expressed nucleolin (green fluorescent spot) and paracancerous area with low-expressed nucleolin. Then, in enlarged image (Fig. 5C), nucleolin merged greatly in cell nucleus with red fluorescence in turmorous area, but no fluorescence signal of nanoprobe and nucleolin in paracancerous area. Importantly, it is found that the red fluorescent of nanoprobe merged perfectly with green fluorescent nucleolin in cancerous tissue, but no apparent red fluorescence in paracancerous tissue (Fig. 5D). Above results suggesting that the nanoprobe could effectively and differentially highlight cancerous tissue in biopsy specimens of patients with invasive ductal carcinoma of the breast.

And then, we also evaluated the expression of NCL in tumor and neighbor normal breast tissues by Western blotting. It is found that most of the tumor tissues exhibit significantly up-regulated NCL levels when compared against neighbor normal tissues (Fig. 5E). Combined with the results of the clinical diagnosis report, the higher expression of NCL showed higher grade malignancy (Fig. 5F), indicating the expression level of NCL is a feasible defining features in human invasive ductal carcinoma of different malignancy grade and can be used in predicting tumor malignancy [47]. In that way, the nonoprobe might be available for distinguishing the malignancy grade of invasive ductal carcinoma in clinic.

### Potential clinical application in tumor grade diagnosis grade

**Fig. 6:**
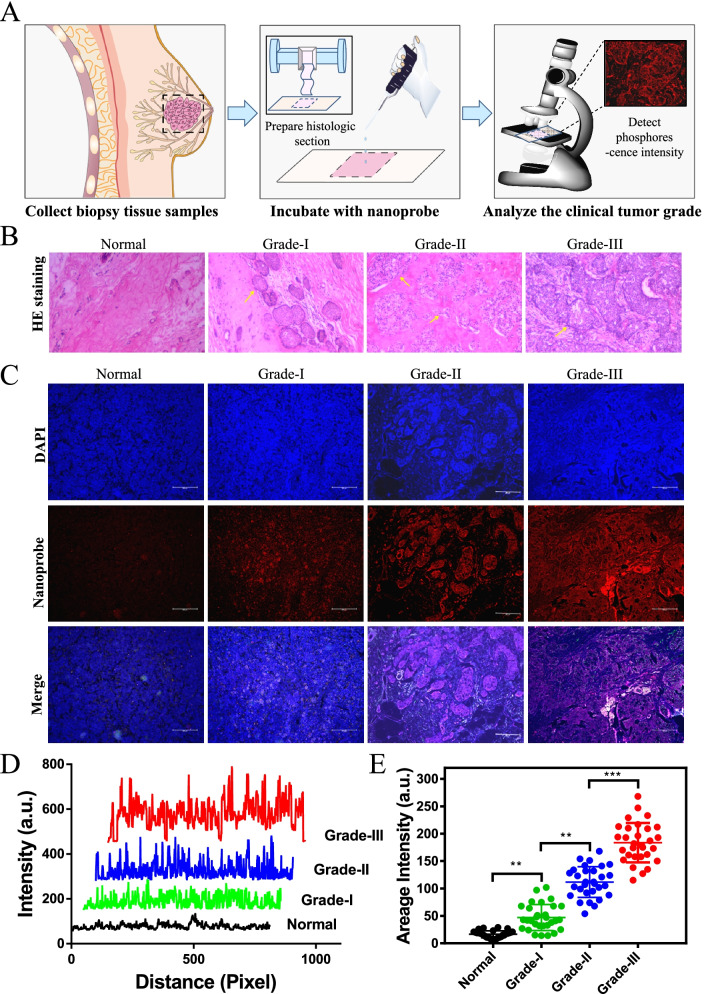
The effectiveness of nanoprobe to distinguish different grades of invasive ductal carcinoma. **A** The operation procedure flow diagram for the nanoprobe to detect tumor tissue specimens. **B** The pathological characteristics of normal and grade I-III tissues of invasive ductal carcinoma specimens by HE staining. Each group of ten specimens. **C** The imaging of nanoprobe (5 μM) for normal and different tumor grade specimens. The paraffin sections were stained with DAPI (blue) and nanoprobe (red). Scale bar: 200 μm (**D**) The emission intensity curve of nanoprobe in different specimens at the trace of white mark line. **E** The statistical analysis of the average intensity of equal area in normal different tumor grade specimens of each group of 10 cases and three repetitions respectively. *n* = 10 *P < 0.05,** *P* < 0.01 and ns—not significant

Then, we used more samples, which are definitely diagnosed and divided into stages of invasive ductal carcinoma, to evaluate the availability of nanoprobe act as a convenient and rapid probe to define the tumor grade through testing the luminescent intensity in biopsy tissue section (Fig. 6A). HE staining showed that the arrangement of normal tissue cells was tight with light red staining, but it revealed the well-defined tumor without obvious invasion to adjacent normal tissue for grade I samples (Fig. 6B). However, with the tumor development to grade II and III, it invades a tissue area as a large number of interlocked tumors and the boundaries between malignant tissue and healthy tissue are blurred and, eventually dissolved.

Moreover, further study shown that the expression levels of NCL in invasive ductal carcinoma are increased in tumor initiation and progression, that it's a critical factor to distinguish tumor grade. It is found that the nanoprobe displayed prominent differences in imaging capability for different grades of invasive ductal carcinoma, the higher degree of malignancy, the higher phosphorescent intensity (Fig. 6C). Particularly, the nanoprobe can significantly highlight tiny focus of invasive ductal carcinoma grade-I patient and show a well-defined tumor area. In these clinical specimens, the nanoprobe emitted extremely weak red signal in normal tissues with intensity at the trace of mark line about 0 ~ 60 a.u., but it is observed quite strong red phosphorescence in grade I-III tissues with intensity at the trace of mark line range of 50–260 a.u. (Fig. 6D), which could obviously differentiate cancer and non-caner area. To further make clear the effectiveness and credibility of the nanoprobe distinguish tumor grade through phosphorescent intensity range, it still need to be improved by expanding the specimens' quantity and extending the number of repeats. Through statistical analysis of five specimens of every group for three repeats, it is found that the average intensity in equal area of nanoprobe in normal tissues is range of 7 ~ 28, in grade I tissues is range of 11 ~ 73, in grade II tissuess is range of 45 ~ 157 and in grade III tissues is range of 94 ~ 224 (Fig. 6E). Compared with common used tumor marker-based methods in clinilical, which is expensive, or the operation is complex, high maintenance costs, is not conducive to the promotion. This study indicated that the synthetic nanoprobe has the potential to act as a convenient and cost-effective diagnostic agent for diagnosis of breast cancer.

## Discussion

Breast cancer is a significant global health problem and leading cause of mortality in women. Once the patient was diagnosed as breast cancer, the establishment of cancer's stage would help to determine the prognosis and best treatment options. Moreover, a biopsy is the only definitive way to make a diagnosis for malignant degree of breast cancer. Owing to the earlier diagnosis to give the earlier treatment is the key point to improve the survival rates of locally advanced breast cancer. Therefore, the discovery of effective tumor targeting imaging tool has important significance for early detection, determination of treatment plan, efficient monitoring of treatment and evaluation of prognosis.

A number of studies indicated that NCL is over-expressed from 3 to sixfold increase in human breast cancer tissues compared to normal breast tissues [27, 28]. Owing to its specific expression on breast cancer cells surface, NCL represents an attractive target for anti-tumor treatment. However, an aptamer AS1411 forms a stable G-quadruplex structure, which exhibited high affinity and specific binding ability to nucleolin. In this regard, AS1411 may function as a NCL specific binding unit to target recognize breast cancer cells. Herein, Ru(II) polypyridyl complexes (RuPEP) with alkynes have excellent luminescent properties, which have been investigated as promising deep-tissue photosensor which can pass through the tissues up to 16 mm thickness when activated by red light after synthesis [24], and it can also be utilized to highlight the nucleus efficiently [25]. Combine the advantage of AS1411 and RuPEP, an effective molecular device for imaging breast tumor cells using nanoparticle probes comprising AS1411 aptamers and Ru (II) complexes that targeting recognize NCL on the cell surface membranes of tumor cells and enter into tumor cell nuclei.

In this study, the nanoprobe emits strong red phosphorescence, which can specifically highlight tumor cells in both in vitro and in vivo models. Further study indicate that the tumor-targeting phosphorescence probe together with a tissue penetrating infrared imaging system may provide immediate clinical benefit by enabling clinicians to track tumor cells that over-express NCL in invasive ductal carcinoma patients. And the results of the clinical diagnosis report, the higher expression of NCL showed higher grade malignancy, indicating the expression level of NCL is a feasible defining features in human invasive ductal carcinoma of different malignancy grade and can be used in predicting tumor malignancy. It is found that the nanoprobe displayed prominent differences in imaging capability for different grades of invasive ductal carcinoma, the higher degree of malignancy, the higher phosphorescent intensity. Moreover, the nanoprobe is easy to operate to rapidly detect the tumor area in the fresh frozen section. In contrast to the diagnosis with paraffin section, accurate rate of diagnosis with frozen section was 96.96% [48]. However, considering the nonspecific accumulation of the nanoprobe in multiple mice organs, the possible risk would limit the future clinical application for in vivo imaging. Compared with common used tumor marker-based methods in clinilical, which is expensive, or the operation is complex, high maintenance costs, is not conducive to the promotion. Therefore, the easy self-assembly leading to a complex formation between targeting aptamer and the red phosphor-RuPEP with its unique optical properties, we expect that the experimental nanoprobe will develop as the early diagnostic agent or the indicator of tumors staging and grading in clinical diagnosis for invasive ductal carcinoma.

## Conclusions

In summary, combination with the excellent tumor targeting ability of AS1411 and great phosphorescence emission of Ru complex (RuPEP) to produce a potent nanoprobe can be used as a convenient and rapid tool to highlight and distinguish tumor cells by targeting recognize NCL in vitro and in vivo. Moreover, the nanoprobe can indicate the tumorous grading and staging in human breast cancer patient pathological section, which provides an effective way for breast cancer diagnostic imaging in clinic.

## Supplementary Information

**Additional file 1. **

## Data Availability

All data generated or analyzed during the current study are included in this. published article (and its supplementary information files).

## Abbreviations

NCL: Nucleolin
ATP: Adenosine triphosphate
AS1411: A nucleolin targeting aptamer
RuPEP: Ru(bpy)_2_(EPPIP)(ClO_4_)_2_
bpy: Bipyridyl
*p*-EPIP: 2-(4-Ethynyl phenyl)-1*H*-imidazo[4,5-*f*][1,10]phenanthroline
G4: G-quadruplex
TEM: Transmission electron microscope
AFM: Atomic force microscope
EDS: Energy-dispersive X-ray spectroscopy
DMEM: Dulbecco’s modified Eagle medium
PBS: Phosphate-buffered saline
FBS: Fetal bovine serum
GFP: Green fluorescent protein
BSA: Bull serum albumin
TBST: Tris-Buffered Saline Tween-20
BCA: Bicinchoninic acid
SDS-PAGE: Sodium dodecyl sulfate polyacrylamide gel electrophoresis
AUC: Analytical ultracentrifugation
CCCP: Carbonyl cyanide m-chlorophenyl hydrazone
Dexy: 2-Deoxy-D-glucose
Olig: Oligomycin
SC: Sedimentation coefficients
DAPI: 4',6-Diamidino-2-phenylindole
NIR: Near-infrared fluorescent
(iv) injection: Intravenous injection
LSCM: Laser scanning confocal microscopy
