# Supplementary material for: Fast detection, a precise and sensitive diagnostic agent for breast cancer
Source: J Exp Clin Cancer Res. 2022 Jun 13;41:201. doi: 10.1186/s13046-022-02393-3 (PMC9190138; doi:10.1186/s13046-022-02393-3)
Supplement: Supplementary file 1 — Additional file 1. [file 13046_2022_2393_MOESM1_ESM.doc]

Fast detection, a precise and sensitive diagnostic agent for breast cancer

Qiong Wu,^1,2^ Chanling Yuan,^1,4^ Ningzhi Liu,^1,4^ Jing Shu,^1,4^ Jiacheng Wang,^1,4^ Liang Zeng,^4^* Hao Zhang^2^, Xicheng Wang, ^2,3^* and Wenjie Mei ^1,3^*

1. School of Pharmacy, Guangdong Pharmaceutical University, Guangzhou 510006, China.

2. The First Affiliation Hospital of Guangdong Pharmaceutical University, Guangzhou 510062, China.

3. Guangdong Province Engineering Technology Centre for Molecular Probe and Bio-Medical Imaging Guangdong Pharmaceutical University, Guangzhou 510006, China

4. Department of Pathology, Guangzhou Women and Children's Medical Center, Guangzhou Medical University, Guangzhou, 510623, China

Corresponding authors: Wenjie Mei, Ph.D. Phone: +86-020-39352122 Email: wenjiemei@gdpu.edu.cn, Xicheng Wang, M.D., Phone: +86-020-39352114 Email: 13902400598@126.com, OR Liang Zeng, Ph.D, Phone: +86-020-81886332 Email: [zlxx03@126.com](mailto:zlxx03@126.com，)

1. EXPERIMENTAL SECTION

1.1 Synthesis of *Λ*-[Ru(bpy)_2_(p-PBE)](ClO_4_)_2_ (RuPEP)

**Figure S1** Micorwave-assisted synthesis route of chiral ruthenium(II) complex **RuPEP**.

1.2 Synthesis of 2-(4-bromophenyl)imidazo[4,5*f*][1,10]phenanthroline (*p*-BrPIP)

2-(4-bromophenyl)imidazo[4,5*f*][1,10] phenanthroline (*p*-BrPIP) was prepared by a similar method as the literatures with some modifications. In general Phenanthroline-5,6-dione (347 mg, 1.6 mmol),  4-Bromobenzaldehyde (294 mg, 1.6 mmol) and ammonium acetate (2.53 g) was dissolved in 20 mL acetic acid and the mixture was irradiated under microwave at 110 ^o^C for 30min. Then 20 mL of water was added and the pH value was adjusted to 7.0 by ammonia water at room temperature. A large number of yellow precipitate were obtained after filtration, and then, it was dried under vacuum. The products were purified silica gel chromatography using ethanol as eluent to give the title compound, yield of 93.2%.

1.3 Synthesis of L-[Ru(bpy)_2_(*p*-BrPIP)](ClO_4_)_2_ (L-1)

L-[Ru(bpy)_2_(*p*-BrPIP)](ClO_4_)_2_ was synthesized following the literature but with some modifications^2^. A mixture of L-[Ru(bpy)_2_(py)_2_][o,o’-dibenzoyl-D-tartrate]∙12 H_2_O (520 mg, 0.4 mmol), p-BrPIP (225 mg, 0.6 mmol) and ethylene glycol (54 mL) was refluxed for 8 h under argon. The cooled reaction mixture was diluted with water. Saturated aqueous ammonium sodium perchlorate solution was added under vigorous stirring, and filtered. The dark red solid was collected and washed with small amounts of water and diethyl ether, then dried under vacuum, and purified by Al_2_O_3_ column chromatography on alumina with acetonitrile/toluene (2 :1 v/v) as eluent. The solvent was removed under reduced pressure and red microcrystals were obtained; yield: 73.0 %. ESI-MS (in CH_3_CN, m/z): 888.6 ([M-ClO_4_]^+^), 789.1 ([M-2ClO_4_-H]^+^), 395.3 ([M-2ClO_4_]^2+^).

1.4 Synthesis of L-[Ru(bpy)_2_(p-PTE)](ClO_4_)_2_  (L-2)

L-[Ru(bpy)_2_(p-PTE)](ClO_4_)_2_ was synthesized following the literatures but with some modifications^2^. In general, L-1 (130 mg, 0.125 mmol) and trimethylsilylacetylene (0.09 mL, 0.625 mmol) was dissolved in dry CH_3_CN (15.0 mL), Pd(PPh_3_)_2_Cl_2_ (3.5 mg, 0.005 mmol), CuI (2 mg, 0.010 mmol) and dry Et_3_N (0.02 mL) were then added under N_2_ atmosphere. The reaction mixture was irradiated by microwaves for 30 min at 140 °C. After filtration and evaporation of the solvent, the residue was purified by flash Al_2_O_3_ column chromatography by using CH_3_CN as elute, yield, 48.2%.

1.5 Synthesis of RuPEP

RuPEP was synthesized following the literatures but with some modifications. L-2 (50 mg, 0.051 mmol) and excessive K_2_CO_3_ (220 mg) were reacted in the mixture of MeOH and tetrahydrofuran (1:1) for 4 h at room temperature. After purification and evaporation of the solvent, the residue was purified, yield, 48.2%.

1.6 The characterization of RuPEP

The structure of RuPEP were characterizated by ESI-MS spectra, ^1^H NMR spectra, ^1^H ^1^H COSY spectra, ^13^C NMR spectra, and CD spectra. ESI-MS (in CH_3_CN, *m/z*): 733.2([M-2ClO_4_-H]^+^), Calculated: 733.15. ^1^H NMR(in DMSO-d_6_, δ/ppm) 9.09 (d, *J*=8.3, H_c_, 2H), 8.88 (d, *J*=8.1 Hz, H_3’_, 2H), 8.84 (d, *J*=8.1 Hz, H_3_, 2H), 8.34 (d, *J*=8.5 Hz, H_j_, 2H), 8.23 (t, *J*=8.0, H_4’_, 2H), 8.12 (t, *J*=8.0, H_4_, 2H), 8.07 (d, *J*=5.3, H_a_, 2H), 7.94 (t, *J*=8.3 Hz, H_b_, 2H), 7.86 (d, *J*=4.9 Hz, H_6’_, 2H), 7.77 (d, *J*=8.5 Hz, H_i_, 2H), 7.65–7.61 (d, *J*=8.3 Hz,H_6_, 2H), 7.61–7.57 (t, *J*=7.2Hz, H_5’_, 2H), 7.36 (ddd, *J*=7.2, 5.7, 1.2 Hz, H_5_, 2H);^13^C NMR (101 MHz, DMSO-d_6_) δ 156.73 (s), 156.52 (s), 151.34 (s), 149.78 (s), 145.06 (s), 137.83 (d, *J*=15.7 Hz), 132.48 (s), 130.26 (s), 129.13–125.46 (m), 126.41 (d, *J*=28.5 Hz), 126.41 (d, *J*=28.5 Hz), 123.83 (d, *J*=128.6 Hz)

1.7 Establishment of tumor xenograft in nude mice

Six-week old female BALB/c nude mice (18–20 g) were purchased from Sun Yet-sen University Experimental Animal Center (Guangzhou). All *in vivo* experiments were performed under the guideline approved by the Guangzhou Institute of Biochemistry and Cell Biology, Chinese Academy of Sciences. To establish tumor models, MDA-MB-231 cells were incubated in culture dishes to a density of 1×10^7^/plate and collected by centrifugation (500g/min, 2 min). They were re-dispersed in 2 mL PBS and 1 mL Matrigel for xenograft implantation. The breast cancer xenograft was implanted in nude mice via subcutaneous injection, on the right leg groin, containing 200 μL MDA-MB-231 cell suspension (1×10^7^ cells per mice). The tumors became visible on the 5th day after injection. When the tumor size reach 100 mm^3^ at 9th day after cells injection, the targeted three mice were intravenously injected with nanoprobe saline at the equivalent RuPEP dose of 20 μM (100 μL), respectively.

In vivo bioimaging.

To assess the tumor-targeting efficacy and image quality of different dose regimens of the nanoprobe *in vivo*, three nude mice with transplanted tumors were treated with pure saline and used as the control group. The targeted three mice were intravenously injected with nanoprobe saline at the equivalent RuPEP dose of 20 μM (100 μL). All animals were monitored by NIR imaging at 0, 6, 12, 24, and 48 h. Tumor nodules and organs (heart, liver, spleen, lung, kidney and brain) were removed at 24 and 48 h, respectively, and *ex vivo* NIR imaging was performed.

2. RSULTS

A

B

C

D


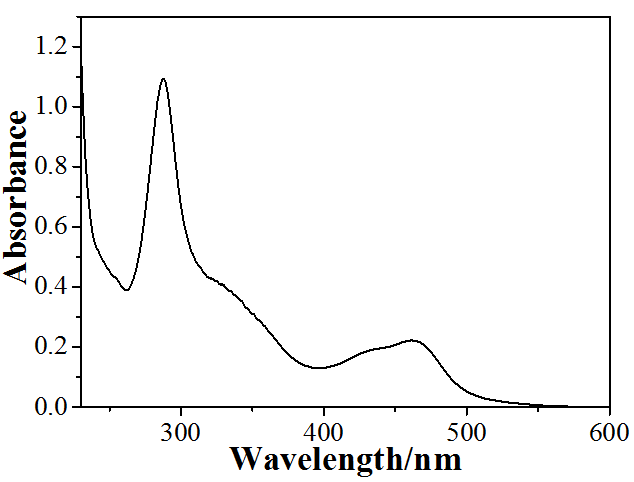


E

**Figure S2**. The characterization data of chiral ruthenium complex RuPEP. (A) The ESI-MS spectra of RuPEP; (B) The ^1^H NMR spectra of RuPEP; (C) The ^1^H ^1^H COSY spectra of RuPEP; (D) The ^13^C NMR spectra of RuPEP; (E) The elctronic absorption spectra of RuPEP (20 μM).


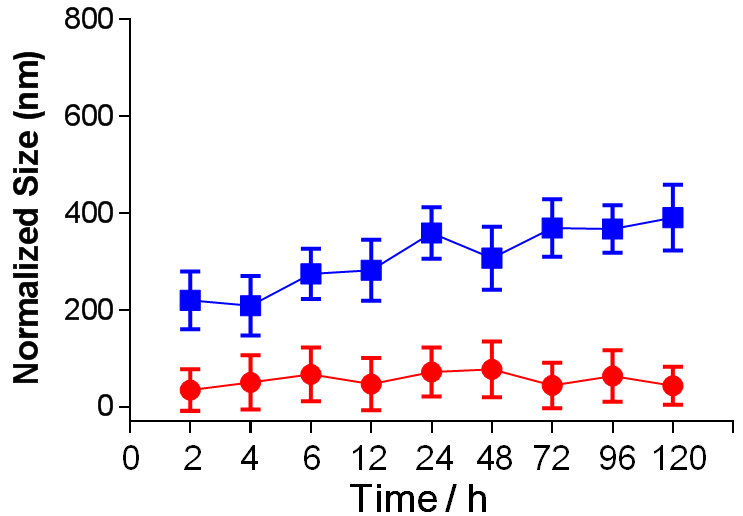


A B

**Figure S3**. (A) The zeta potential of free AS1411 (5 μM), free RuPEP (5 μM) and nanodevice (5 μM). (B) The stability of AS1411 (5 μM) and AS1411@RuPEP (5 μM) in PBS solution.


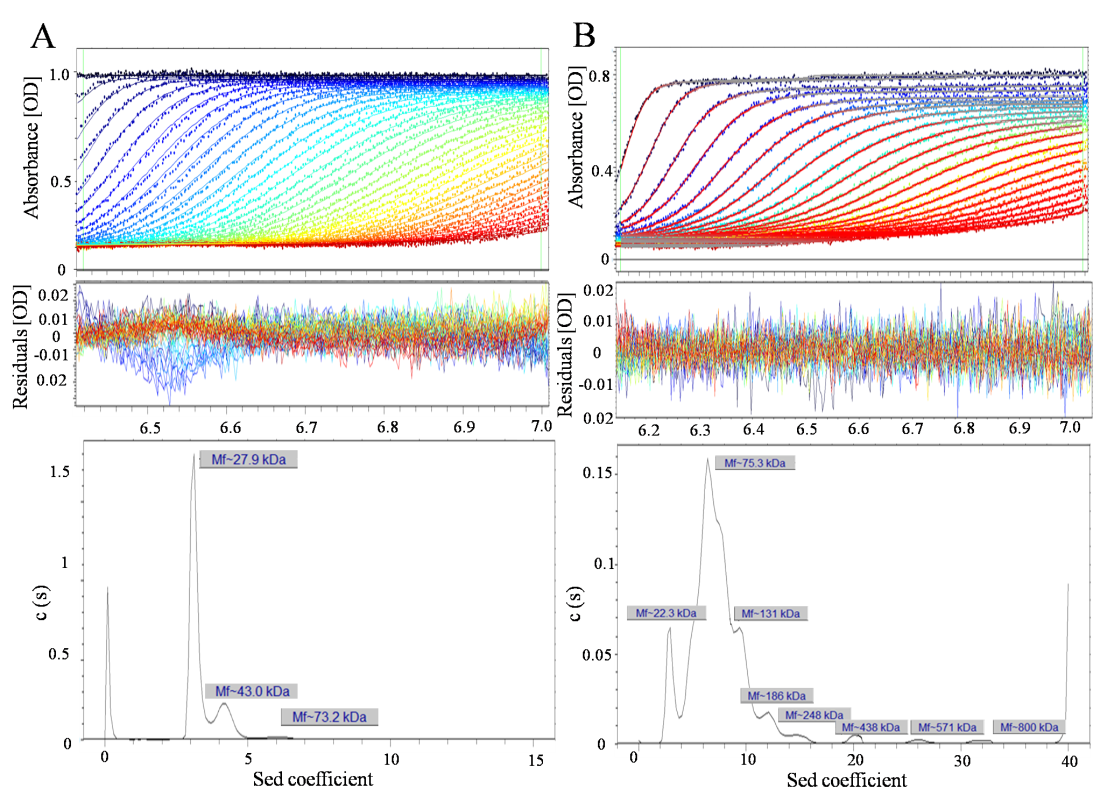


**Figure S4.** Analytical ultracentrifugation analyzes the sedimentation velocity (SV) of AS1411 (A) and AS1411@RuPEP (B). All theoretically calculated SV concentration proﬁles, s (r, t), are generated using SEDFIT, fitting error (a and b), SV data (after wiping off the system noise), and fitting curve (c and d). Sedimentation coeﬃcient distributions c(s) from the analysis of the sedimentation proﬁles of simulated AS1411@RuPEP bound DNA system (e and f), and SV runs monitored by absorbance at 300 nm. Concentrations are [AS1411] = 100 μM, [AS1411@RuPEP] = 100 μM.


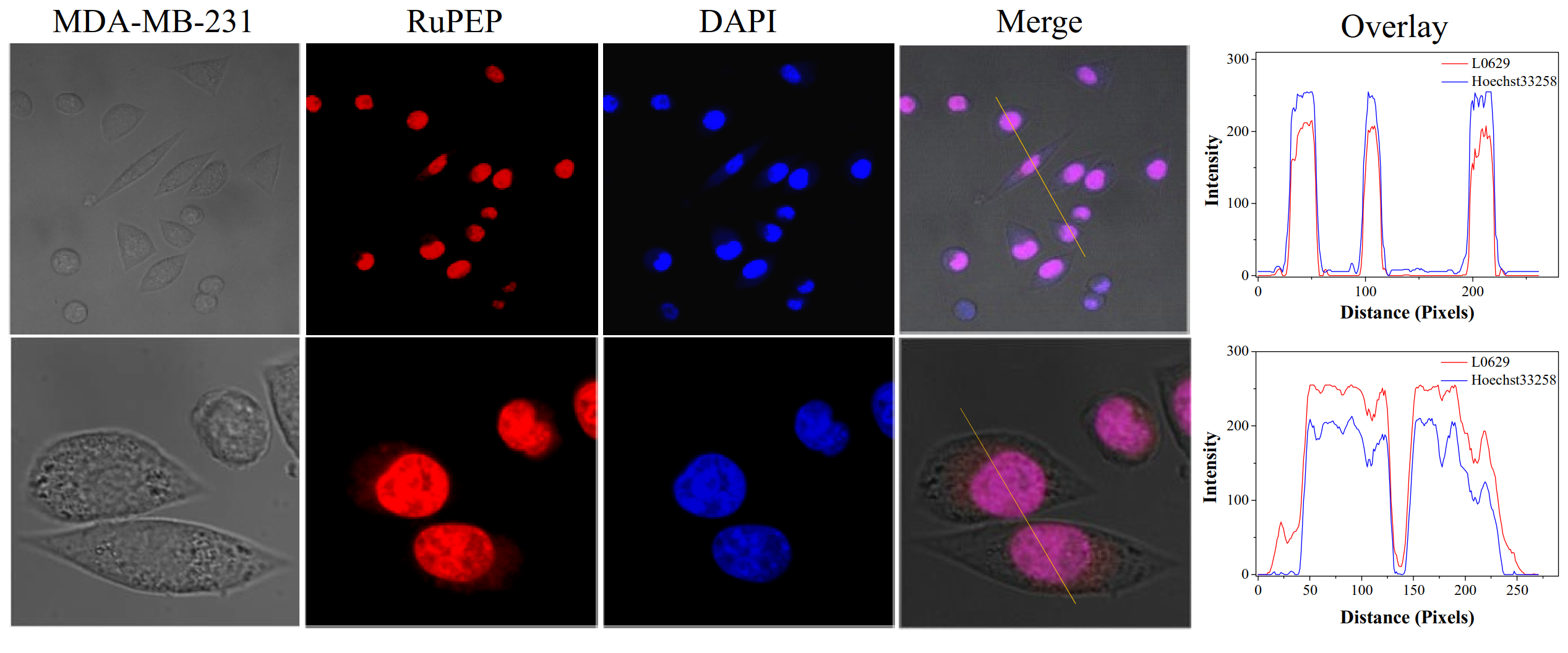


**Figure S5**. The cellular localization of RuPEP in MDA-MB-231 cells. The cells were incubated by RuPEP (5 μM) for 6 h**,** then cells were washed 3 times by PBS and stained with DAPI.


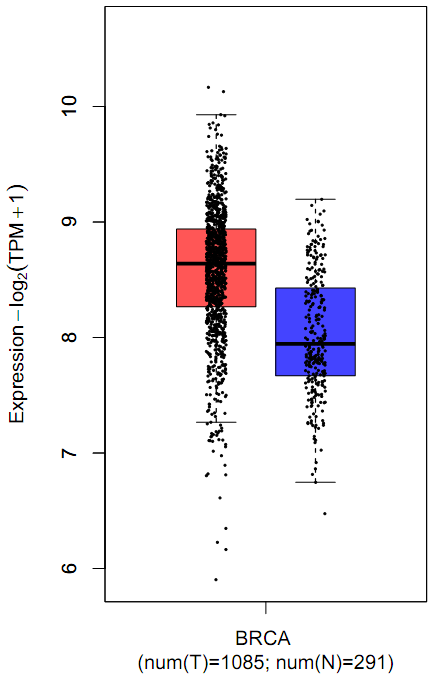

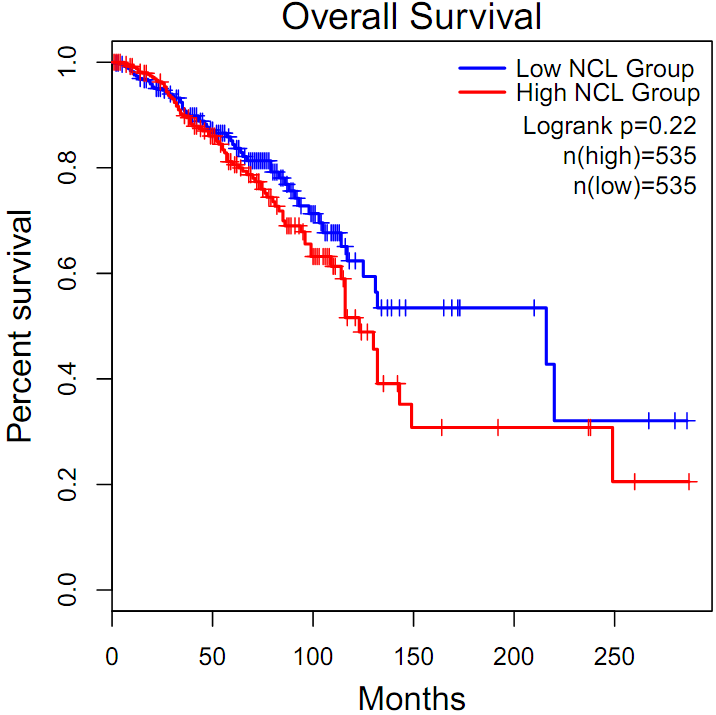

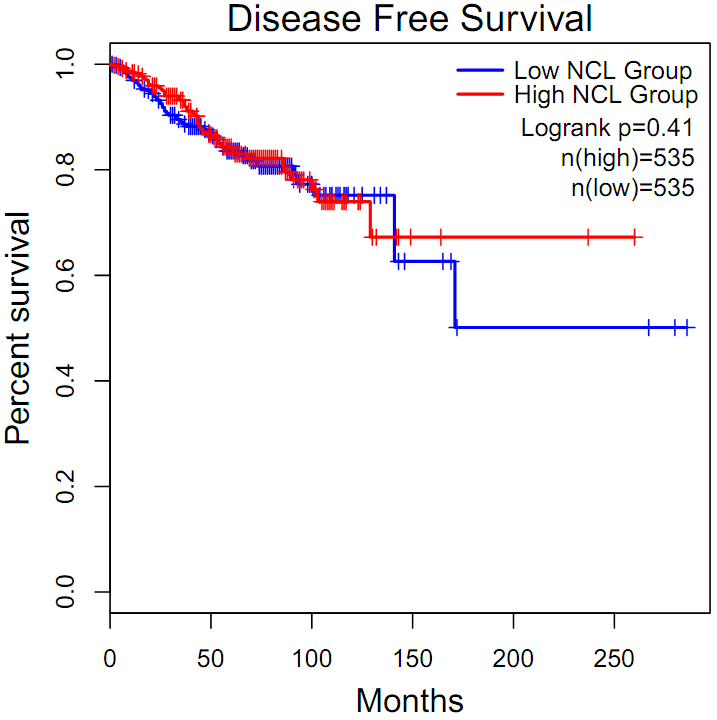


A B C

**Figure S6**. Prognostic power and expression level of NCL in breast cancer. (A) The mRNA expression of NCL was analyzed via GEPIA2. The red and blue boxes represent tumors and normal samples, respectively. Kaplan-Meier Overall Survival (B) Disease Free Survival (C) curve obtained GEPIA2 database stated that BRCA patients with higher expression of NCL had a significantly shorter survival time.


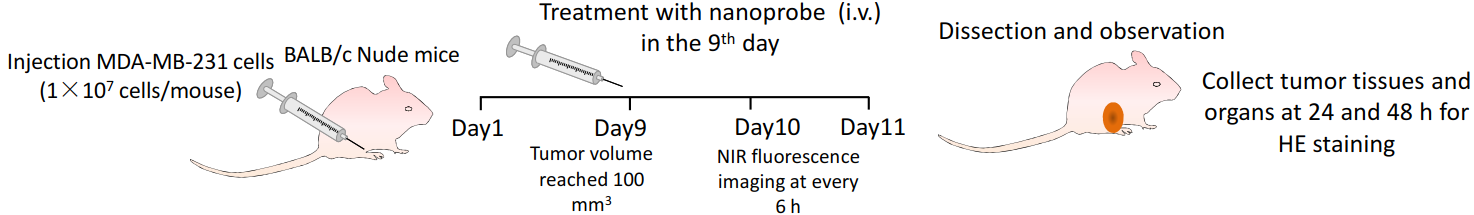


**Figure S7**. The schedule of saline and AS1411@RuPEP (20 μM) treated mice with MDA-MB-231 xenograft tumors.

In vivo Imaging tumor cells

After confirming that the probe selectively binds and translocates as expected to breast cancer cells in culture, we investigated its performance *in vivo* in MDA-MB-231 tumor-bearing BALB/c mice. Specific tumor-targeting images were obtained from nanoprobe interrogated at different injection time points (**Figure S8**). Mice that were imaged in NIR before injection of the probes showed virtually no signal. NIR phosphorescence became visible immediately after intravenous (iv) injection in the tail vein due to the rapid distribution of the probes [43]. Tumor areas were well deﬁned in the mice within the ﬁrst 6 h as the nanoprobe rapidly recognizes and binds its NCL targets in tumor tissues through the enhanced permeability and retention effect (EPR effect) (**Figure S8A**). At 12 h, the illuminated tumor area had increased due to the retained signal from the tumor site, augmented by interference in the ﬂuorescence background from normal tissue. With increasing time, ﬂuorescence from normal tissues, originating from clearance pathways and non-specific uptake, caused the tumor area to become less well deﬁned (**Figure S8B**). By contrast, strong fluorescence from the non-targeting probe RuPEP was observed in the entire mouse within 6 h, that indicates that free RuPEP rapidly distributes in the entire body and increases with time (**Figure S9**). The above results suggest that the constructed nanoprobe selectively and rapidly define tumor tissues after systemic administration with 6 h. Eventually, nanoprobe distributes throughout the entire body, but nevertheless predominately accumulates in tumors. Ex vivo images of organs and tumors taken at autopsy from experimental animals showed that the probe retention in tumors taken at 24 and 48 h were comparable (**Figure S8C**). These data conﬁrm the long tumor retention time of nanoprobe. The ex vivo image after dissection shows that the quantitative distributions of nanoprobe and their non-targeting RuPEP component were determined by fluorescence intensity in the different organs. Signals for the two probes arising in brain tissues were markedly higher at 24 h than at 48 h, while the signals in the kidney were signiﬁcantly lower at 24 h than at 48 h (**Figure S8C**). This indicates that the two probes transport across the Blood-Brain-Barrier and are cleared from the body through kidney ﬁltration. Low metabolism and slow kidney clearance produced high tumor accumulation with greater tumor uptake and stronger fluorescence of the nanoprobe at 24 h than 48 h [44]. The consistency of these data showing the accumulation of the probes in the tumor and kidney from the *in vivo* measurements and after organ extraction clearly indicates that noninvasive real-time in vivo imaging for localizing specific tumors is feasible in spite of renal clearance of these probes (**Figure S8D**). This work demonstrates that nanoprobe can specifically and rapidly image tumor tissues *in vivo,* but the potential application of nanoprobe for *in* [*vivo*](javascript:;) [detection](javascript:;) is limited severely by aggregated distribution in different organs.


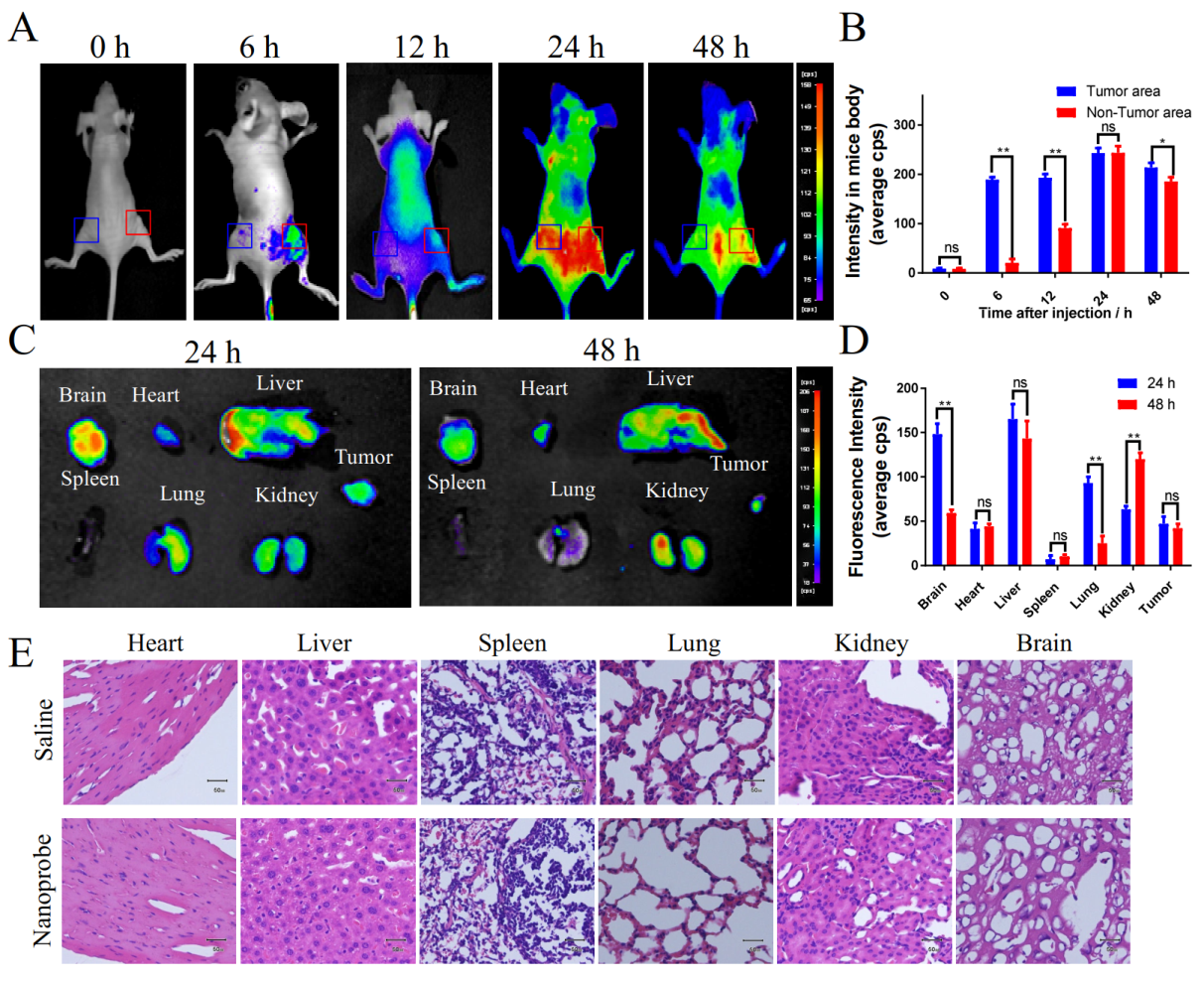


**Figure S8**. Speciﬁc-targeted NIR ﬂuorescence tumor imaging in vivo. (A) The arrows show the tumor sites. Time-dependent in vivo NIR ﬂuorescence images of nude mice bearing MDA-MB-231 cell-derived tumors (S.C. xenograft model) after intravenous injection of 20 μM, 100 μL, AS1411@RuPEP. (Excited by 475 nm, emission at 680 nm). (B) Fluorescence intensity of AS1411@RuPEP in mice tumor area and non-tumor area is quantitatively determined. (C) Tissue distribution and drug metabolism of AS1411@RuPEP at 24 and 48 h. (D)Fluorescence intensity of AS1411@RuPEP (average cps) in dissected organs or tissues is quantitatively determined. Data are presented as the mean ±SD (n = 3). *p < 0.05, **p < 0.01. (E) Histochemistry analysis of heart, liver, spleen, lung, kidney and brain section stained with hematoxylin eosin of kunming mice 24 h after i.v. administration of saline and 50 mg/kg nanoprobes for 7 days, one dose per day. Bar: 100 μm.


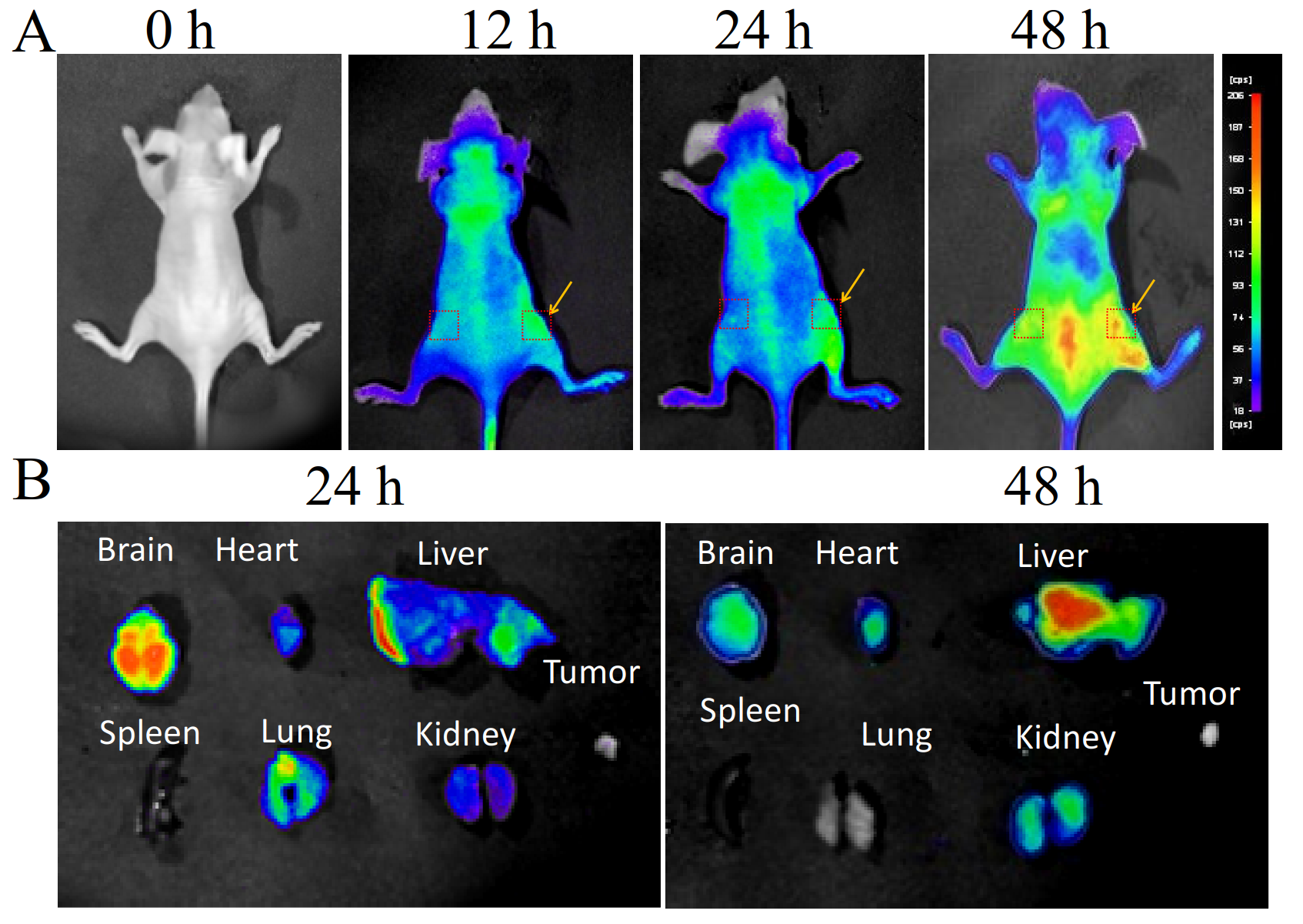


**Figure S9**. (A) The arrows show the tumor sites. Time-dependent *in vivo* NIR ﬂuorescence images of nude mice bearing MDA-MB-231 cell-derived tumors (S.C. xenograft model) after intravenous injection of 20 μM, 100 μL, free RuPEP. (Excited by 475 nm, emission at 680 nm). (B) Tissue distribution and drug metabolism of free RuPEP at 24 and 48 h.


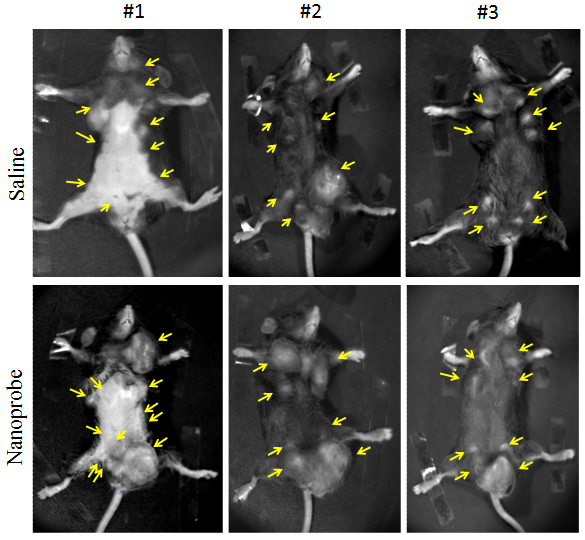


**Figure S10**. Six MMTV-PyMT mice (24 weeks old). Yellow arrows indicate primacy tumor area.


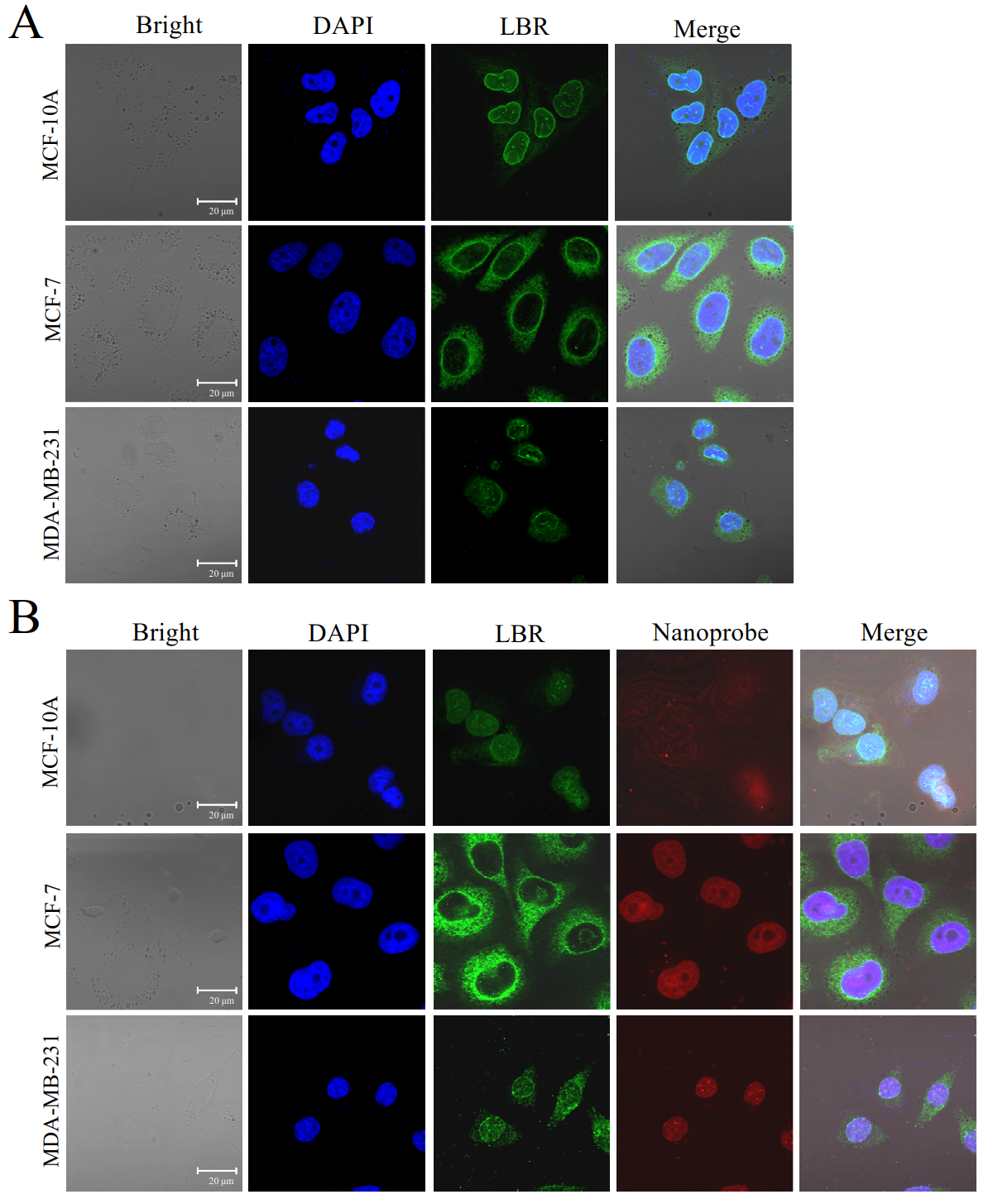


**Figure S11.** (A) The distribution and expression of LBR in breast cancer MDA-MB-231, MCF-7 cells and human normal MCF-10A cells. (B)The localization of AS411@RuPEP nanoprobe in breast cancer MDA-MB-231, MCF-7 cells and human normal MCF-10A cells.

**Figure S12**. The influence of body weight of kunming mice (n= 3) by nanoprobe in treatment days (1-8 days).


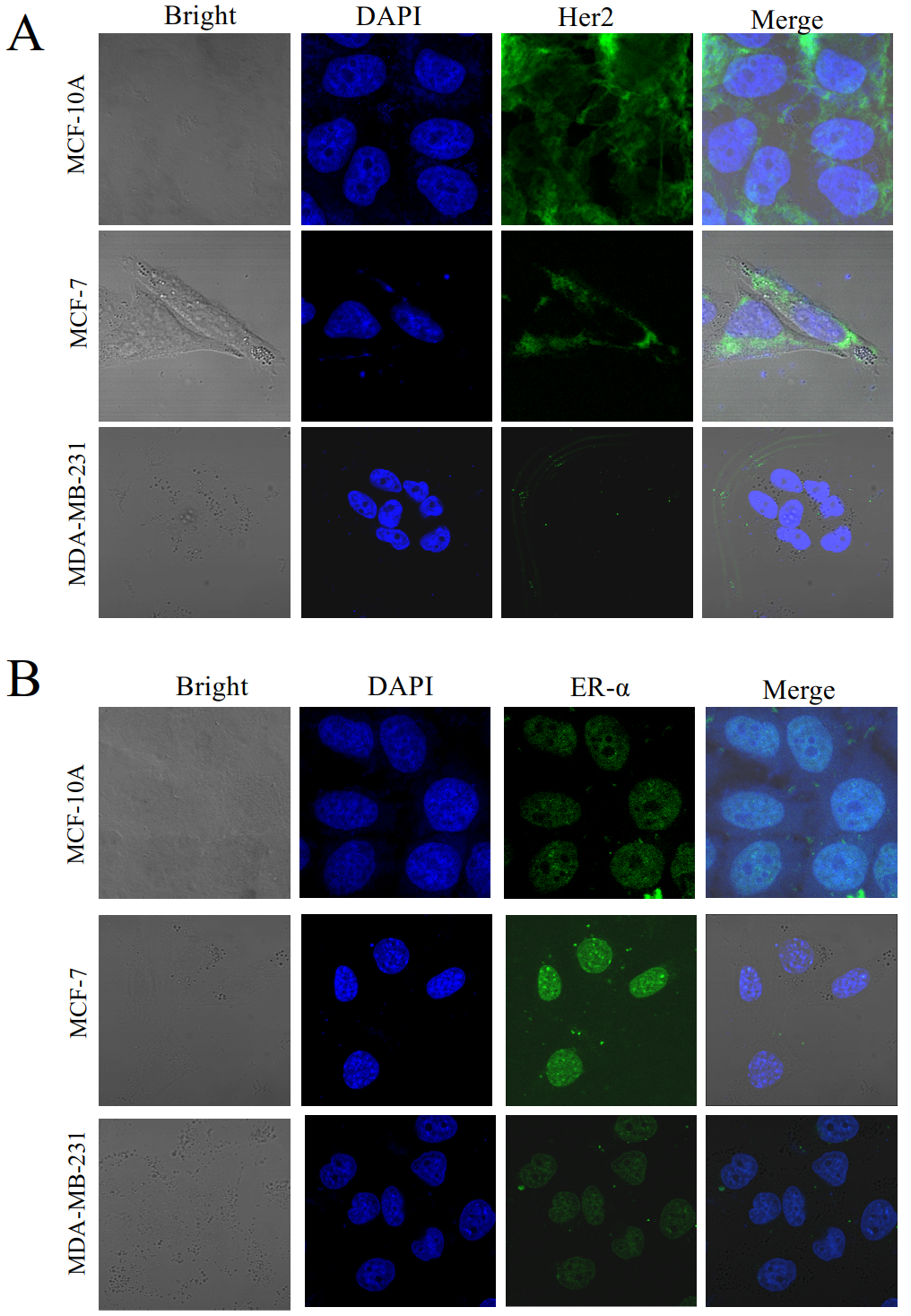


**Figure S13.** The distribution and expression of Her2 (A) and ERα (B) in breast cancer MDA-MB-231, MCF-7 cells and human normal MCF-10A cells. The localization.


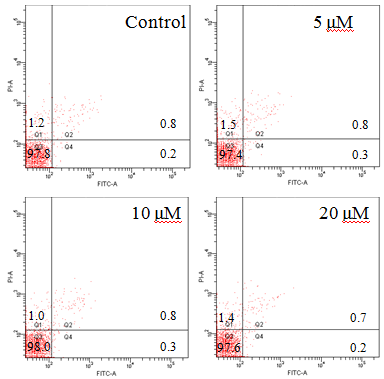


A B

**Figure S14**.The toxicity assessment of nanoprobe in vitro. (A) In vitro cell viabilities of MDA-MB-231 and HaCaT cells incubated with nanodevice at 37 ^o^C for 72 h. (B) The apoptosis induction of MDA-MB-231 cells induced by nanodevice (0, 5, 10 and 20 μM) at 37 ^o^C for 72 h.
